# Effect of Laser Processing Parameters on the Quality of Titanium Alloy Cladding Layer on Carbon Fiber-Reinforced Polymer

**DOI:** 10.3390/polym17091195

**Published:** 2025-04-27

**Authors:** Jiayan Li, Xuan Su, Fenxiang Wang, Donghe Zhang, Yingke Wang, Haoran Song, Jie Xu, Bin Guo

**Affiliations:** 1 CNOOC Refining & Petrochemicals Co., Ltd., Beijing 100010, China; 2 Zhengzhou Research Institute, Harbin Institute of Technology, Zhengzhou 450046, China; zhangdonghe@hit.edu.cn (D.Z.); wangyingke66@hit.edu.cn (Y.W.); xjhit@hit.edu.cn (J.X.); 3 School of Materials Science and Engineering, Harbin Institute of Technology, Harbin 150080, China; guobin@hit.edu.cn; 4 Key Laboratory of Micro-Systems and Micro-Structures Manufacturing of Ministry of Education, Harbin Institute of Technology, Harbin 150080, China

**Keywords:** carbon fiber-reinforced polymer, laser cladding, TC4 coating, microstructure, shear strength

## Abstract

To address the insufficient bonding performance between TC4 (Ti-6Al-4V) coating and carbon fiber-reinforced thermoplastic (CFRP) matrices that limits engineering applications of composite structures, TC4 coatings were fabricated on CFRP polymer composites via laser cladding and analyzed using scanning electron microscopy (SEM) and transmission electron microscopy (TEM) to examine the interface morphology, microstructure, and phase composition. The influence of laser processing parameters on the cladding quality was assessed based on the mechanical performance of the TC4 coating. The findings revealed that insufficient laser power (<230 W) or excessive scanning speed (>1.4 m/min) led to incomplete melting of TC4 powder, preventing the formation of intermetallic compound (IMC) layers. Conversely, excessive laser power (>270 W) or a low scanning speed (<1.0 m/min) caused thermal decomposition of the CFRP due to its limited thermal resistance, leading to interfacial defects such as cracks and pores. The interface between the CFRP and TC4 coating primarily comprised granular TiC and acicular α′ martensite, with minor TiS_2_ detected. Optimal mechanical performance was achieved at a laser power of 250 W and a scanning speed of 1.2 m/min, yielding a maximum interfacial shear strength of 18.5 MPa. These findings provide critical insights for enhancing the load-bearing capacity of TC4/CFRP aeronautical composites, enabling their reliable operation in extreme aerospace environments.

## 1. Introduction

The carbon fiber-reinforced thermoplastic (CFRP) composites have gained progressive application in aerospace and petrochemical industries owing to their exceptional specific strength-to-weight and stiffness-to-weight ratios, corrosion resistance, and fatigue/fracture durability [1,2,3]. These materials must satisfy stringent operational requirements in extreme environments, including (1) high-temperature tolerance with flame-retardant properties; (2) erosion resistance against airborne particulates such as water droplets, sand, and mineral debris; and (3) sufficient electrical conductivity to ensure lightning strike protection for aircraft systems [4]. Therefore, the development of protective coatings for CFRP composite materials is crucial for meeting the functional and protective requirements of aerospace and petrochemical industry components [5].

Surface coatings for CFRP composites primarily consist of five categories: metallic coatings (e.g., titanium alloys, aluminum) demonstrating metallurgical bonding capabilities [6], ceramic coatings [7] (e.g., SiC, Al_2_O_3_) with high thermal stability, polymer-based coatings [8] (e.g., polyimide, PEEK) offering chemical compatibility, composite coatings [9] (e.g., Ti/SiC gradient systems) combining multiple material advantages, and functional coatings [10] (e.g., graphene-enhanced conductive layers, diamond-like carbon wear-resistant films) providing specialized surface properties. In aerospace applications, titanium alloy coating systems have attracted significant research interest due to their exceptional strength-to-weight ratio and capacity for fabricating ultrathin protective layers while maintaining structural integrity [11]. This characteristic proves particularly advantageous for aircraft components requiring strict weight optimization. However, the low thermal resistance of CFRP composites requires strict temperature control at the matrix surface during titanium alloy protective coating preparation [12].

Current techniques for depositing titanium alloy coatings on CFRP composites—including physical vapor deposition (PVD), chemical vapor deposition (CVD), electroplating, thermal spraying, and cold spraying—all exhibit specific limitations [13,14,15,16]. PVD and CVD require substantial capital and operational expenditures while restricting workpiece dimensions due to their atomic-scale deposition mechanisms [17,18,19]. Electroplating faces challenges such as weak coating adhesion, performance instability, and environmental hazards [20,21]. Though spraying methods provide operational simplicity and material flexibility, they suffer from inadequate bonding strength and porosity issues [22,23]. In summary, the main problems are as follows: First, insufficient coating adhesion stems from material heterogeneity-induced interfacial stress concentrations, where coatings form mechanical rather than metallurgical bonds with CFRP substrates. Second, significant thermal expansion coefficient mismatches generate excessive interfacial stresses that further degrade adhesion. Third, CFRP’s inherent thermal sensitivity and mechanical softness lead to substrate deformation or damage during coating processes. Addressing these challenges necessitates developing a titanium alloy coating process that ensures robust interfacial adhesion while minimizing substrate damage, a critical requirement for advancing CFRP applications in engineering fields. Laser cladding coating technology has been developed and applied due to its advantages of low heat input, protecting the substrate, high bonding strength, superior coating performance, flexible processing, and reduced material waste [24,25]. Related studies [26] have examined the formation mechanism and interface stress in laser-cladded titanium alloy coatings. However, current research lacks systematic optimization of critical laser parameters (e.g., power density, scanning speed, spot overlap). Therefore, it is an urgent research priority to explore a laser cladding process for preparing a surface titanium alloy coating on CFRP composite material with high strength and minimal damage to the matrix.

In this study, Ti alloy coatings were deposited on CFRP composites via laser cladding, and the effects of laser processing parameters on coating quality were systematically investigated. The objective was to address the challenges associated with Ti alloy coating deposition on CFRP composites, thereby providing both theoretical insights and technical advancements for improving the bonding performance of Ti alloy coating on CFRP substrates.

## 2. Materials and Methods

### 2.1. Materials

**Figure 1:**
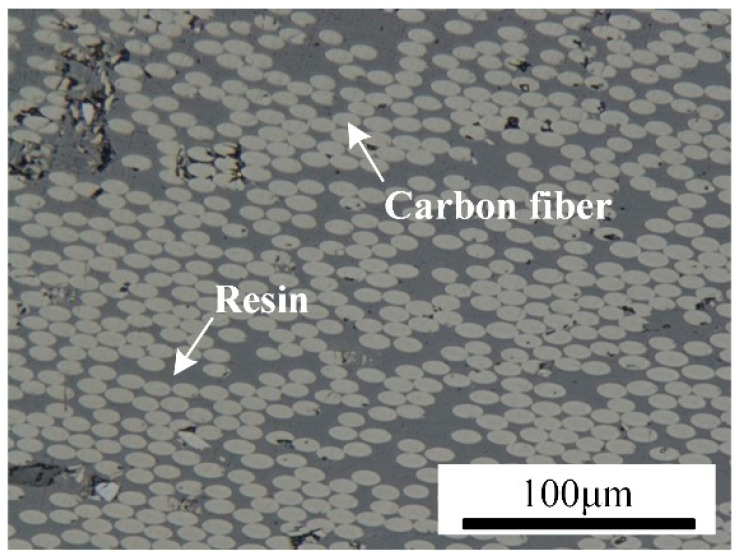
Macroscopic morphology of CFRP.

The materials used in this study include CFRP composites and TC4 alloy powder. The CFRP consists of polyphenylene sulfide (PPS) as the matrix, with a carbon fiber volume fraction of 50% and a thickness of 2.5 mm. The metallographic macroscopic appearance of the CFRP is shown in Figure 1.

**Figure 2:**
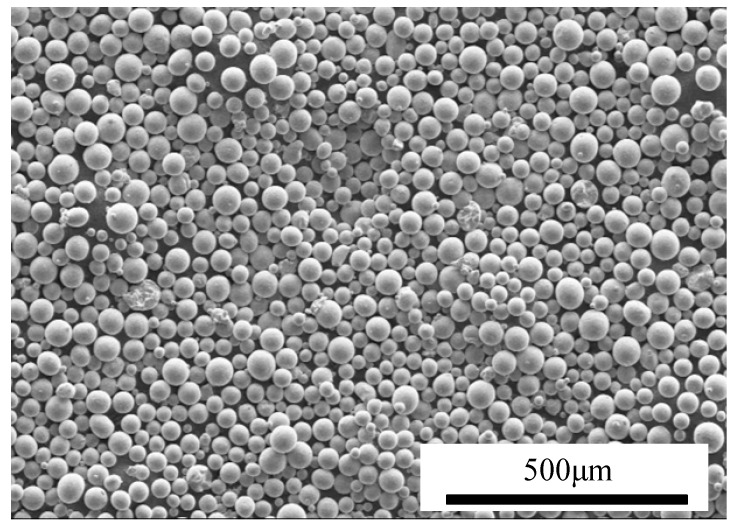
SEM micromorphologies of TC4 powder.

Prior to deposition/cladding, the CFRP sheets were cut into rectangular samples (50 mm × 15 mm × 2.5 mm) using wire electrical discharge machining (EDM) and subsequently ultrasonically cleaned in anhydrous ethanol to remove surface contaminants such as oil and debris. For laser cladding, spherical TC4 alloy powder (from AVIMETAL AM, Beijing, China) was employed, with a particle size distribution ranging from 45 to 75 μm, as shown in Figure 2. The chemical composition of the TC4 powder is detailed in Table 1.

### 2.2. Methods

The laser cladding parameters employed in this study for TC4 coating preparation were established through prior experimental optimization. Specifically, four laser power settings (210 W, 230 W, 250 W, and 270 W) and four scanning speed values (1.0 m/min, 1.2 m/min, 1.4 m/min, and 1.6 m/min) were systematically selected as the core process variables. To prevent direct laser irradiation from causing severe damage to the CFRP matrix, the pre-positioned powder method was employed. In this approach, TC4 powder was pre-deposited onto the CFRP surface before laser exposure. The laser energy initially acted on the TC4 powder, and the heat was subsequently transferred to the CFRP-TC4 interface via thermal conduction, effectively minimizing the risk of CFRP degradation.

**Figure 3:**
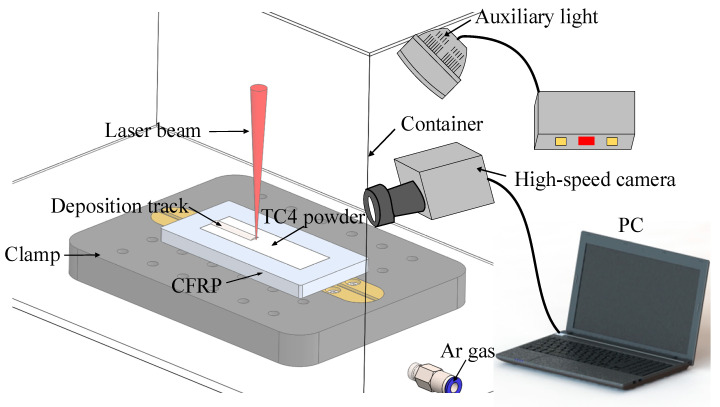
Schematic of laser cladding process.

The schematic of the laser cladding system is illustrated in Figure 3. The process was performed using an IPG YLR-5000 fiber laser (IPG Photonics, Burbach, Germany) with the following parameters: wavelength of 1070 nm, beam waist radius of 0.45 mm, Rayleigh length of 14.97 mm, and a defocused spot diameter of 3.7 mm achieved by adjusting the defocusing distance to 60 mm. The laser beam has a Gaussian spatial energy distribution and a single-mode beam quality (M^2^) < 1.1. The pre-deposited titanium alloy powder layer had a controlled thickness of 0.7 mm. To prevent oxidation at elevated temperatures, laser cladding was performed in an argon-filled chamber. The stability of the cladding process was continuously monitored in real time using a high-speed camera system, enabling detailed observation of the melting zone characteristics and overall process stability.

### 2.3. *Microstructural and Mechanical Characterization*

Prior to CFRP/coating interface characterization, pretreatment procedures were systematically conducted. Specimens from the interfacial region were sectioned using an internal circular cutting machine and cold-mounted for structural preservation. Sequential grinding was performed with 120#, 200#, 400#, 600#, and 800# metallographic sandpapers, followed by mechanical polishing using 2.5 μm and 1.0 μm diamond suspensions to achieve mirror-like surfaces. Chemical etching with 3% HF + 30% HNO_3_ + 67% H_2_O solution (10 s exposure) preceded microstructural analysis via scanning electron microscopy (Quanta 200FEG, FEI, Hillsboro, OR, USA). For FIB-TEM preparation, laser-clad CFRP/TC4 samples (50 mm × 15 mm × 2.5 mm) were mechanically polished and adhered to an Al alloy tray using conductive silver adhesive. A protective Pt layer (8 μm × 2 μm × 1 μm) was electron-beam deposited prior to focused ion beam (FIB) processing. The sample stage was tilted to 7°, and a 1.5–2 μm thick lamella was extracted using an Omniprobe manipulator (Oxford Instruments, Oxford, UK). Subsequent FIB milling at 1 nA current enabled cantilever separation, followed by Cu grid mounting and ion thinning to electron transparency. Final characterization employed transmission electron microscopy (Tecnai G2 F30, FEI, Hillsboro, OR, USA) for high-angle annular dark-field imaging and elemental distribution analysis.

**Figure 4:**
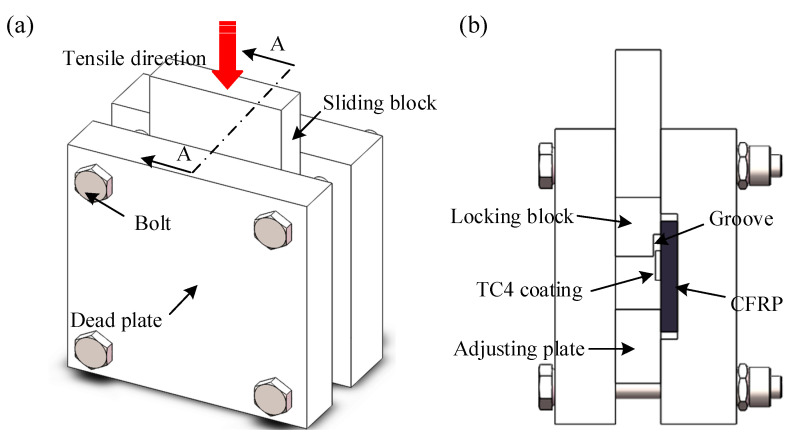
Schematic of the compression shear test for interface performance: (**a**) isometric side view, (**b**) sectional view.

To precisely determine the bonding strength at the CFRP-coating interface, a custom-designed fixture was utilized to secure the specimens during mechanical testing in Figure 4. In the compression shear test, an electronic universal testing machine (MICROTEST2000, Gatan, Pleasanton, CA, USA) applied force to a slider at a constant speed of 0.5 mm/min. For each set of processing parameters, three shear test specimens were selected for measurement.

## 3. Results and Discussion

### 3.1. Effect of Laser Power on the Quality of TC4 Cladding Layer

The cladding process was recorded using a high-speed camera system to analyze the evolution of the cross-sectional morphology of the TC4 coating on CFRP under varying laser power and scanning speed. The impact of laser power on the cladding process was assessed through image analysis from the high-speed recordings.

**Figure 5:**
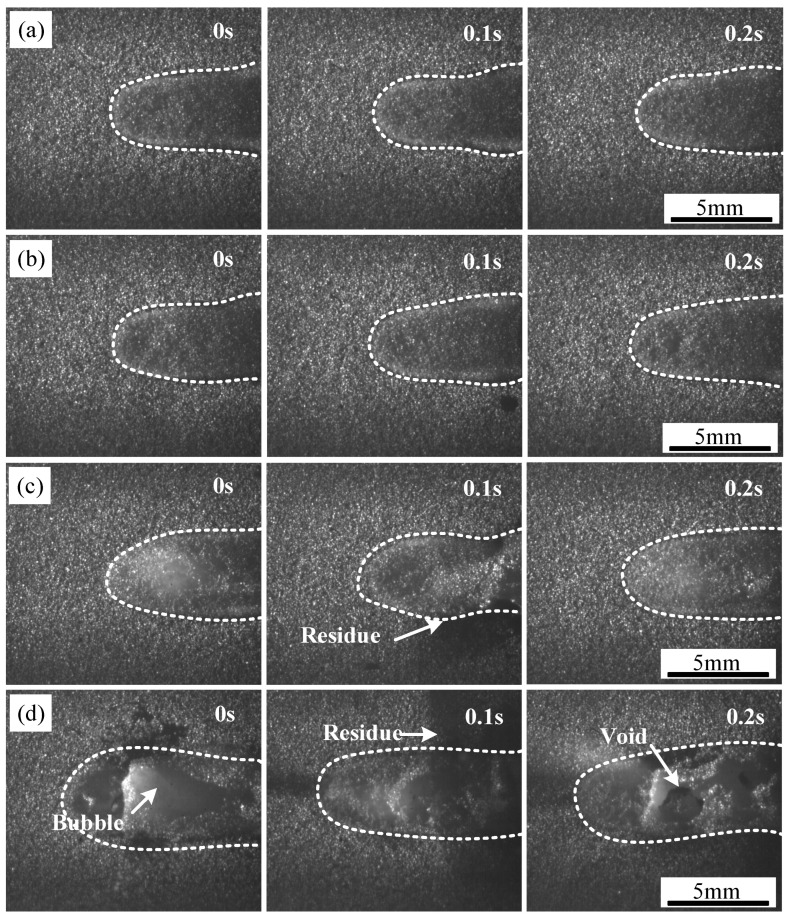
Melting zone of coating with different laser powers: (**a**) P = 210 W, (**b**) P = 230 W, (**c**) P = 250 W, and (**d**) P = 270 W.

At a scanning speed of 1.2 m/min, the TC4 coating formed at different laser power levels is shown in Figure 5, with each frame captured at 0.1 s intervals. The contour of the powder melting zone is highlighted with a white dashed line. At lower laser power levels (P = 210 W and 230 W), the cladding process was stable, with a smooth coating surface. However, as the laser power increased, black solid residues appeared near the coating at 250 W. These residues resulted from the thermal decomposition of PPS, which released gases that escaped through gaps in the pre-positioned powder and molten zone before cooling and adhering to the coating surface.

When the laser power was further increased to 270 W, a more pronounced presence of black solid residues, large bubbles, and surface defects such as pores was observed. This behavior was attributed to the continued thermal decomposition of PPS, which released a considerable volume of gas that escaped through the molten TC4 layer. The accumulation of these gases led to the formation of bubbles, which, upon collapse, left voids in the coating. Additionally, an increase in power caused a higher proportion of the liquid phase in the molten pool, promoting the absorption of surrounding powder. This effect contributed to spheroidization, where the molten metal, influenced by surface tension and gravitational forces, failed to spread uniformly and instead formed isolated spherical particles. The presence of spheroidized particles resulted in increased surface roughness and porosity, ultimately compromising the mechanical performance of the coating. Consequently, an excessive laser power level led to an uneven TC4 coating on the CFRP surface, characterized by roughness, porosity, and poor interfacial integrity.

**Figure 6:**
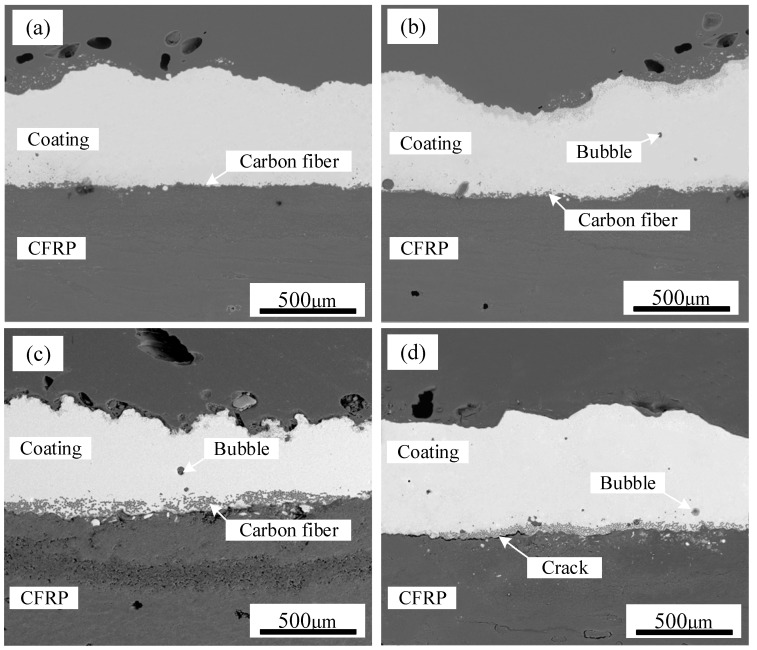
The cross-section of CFRP/TC4 under different laser powers: (**a**) P = 210 W, (**b**) P = 230 W, (**c**) P = 250 W, (**d**) P = 270 W.

Figure 6 presents SEM images of TC4 coating deposited on CFRP at different power levels. In backscattered electron mode, materials with higher atomic numbers appear brighter, with the TC4 metal coating displaying a bright white contrast, while the CFRP matrix appears in dark gray. The results indicate that the TC4 powder exhibited good wettability on the CFRP surface, forming a dense, uniform, and continuous coating. However, during laser cladding, PPS decomposition generated gases that interacted with the pre-positioned coating powder, leading to surface irregularities and non-uniform coating deposition. As the laser power increased from 210 W to 270 W, a continuous coating was successfully formed on the CFRP substrate. However, the spread distance of the TC4 coating over the CFRP surface increased with higher power, leading to greater incorporation of carbon fibers into the TC4 layer. The increased heat input also intensified PPS decomposition, generating larger gas volumes and increasing the number of air voids within the TC4 coating. At excessive power levels, cracks appeared at the CFRP/TC4 interface, as illustrated in Figure 6d, indicating a reduction in coating adhesion due to increased thermal stress.

### 3.2. *Effect of Laser Scanning Speed on the Quality of TC4 Cladding Layer*

**Figure 7:**
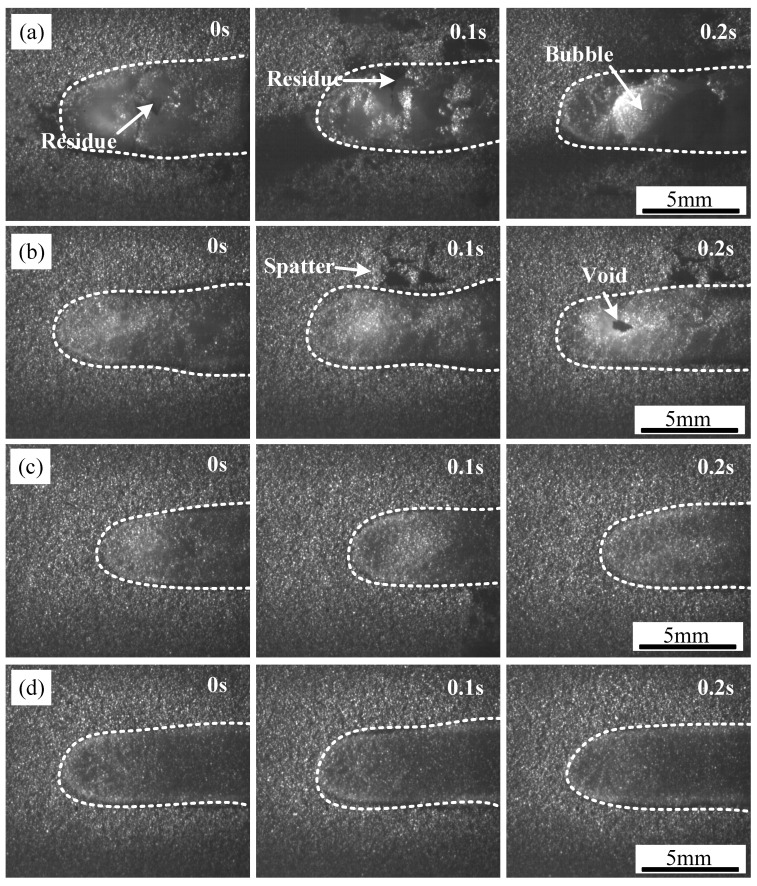
Melting zone of coating with different scanning speeds: (**a**) v = 1 m/min, (**b**) v = 1.2 m/min, (**c**) v = 1.4 m/min, (**d**) v = 1.6 m/min.

The influence of scanning speed on the TC4 coating process during laser cladding was investigated under a laser power of 250 W. The results, presented in Figure 7, illustrate the evolution of the coating morphology at different scanning speeds, with each frame captured at 0.1 s intervals. The contour of the powder melting zone is highlighted using a white dashed line. At a low scanning speed of 1 m/min, a significant amount of black solid residue and large bubbles appeared on the surface of the cladding layer, as shown in Figure 7a. However, when the scanning speed increased to 1.6 m/min, the cladding process stabilized, resulting in a smooth coating surface free of noticeable fluctuations, as observed in Figure 7c,d. The instability in the laser cladding process was mainly concentrated at the center of the laser action zone. One of the primary reasons for this phenomenon is the unstable behavior of the molten pool surrounding the liquefied TC4. Under the influence of the laser, particularly at high energy densities, intense evaporation of Ti alloy powder occurs. This leads to the generation of substantial metal vapor, which disturbs the molten pool and causes spattering, as indicated in Figure 7b. Additionally, the thermal decomposition of CFRP at the interface generates gases, further contributing to pronounced fluctuations in the pre-coated layer, thereby affecting the overall uniformity of the deposition process.

**Figure 8:**
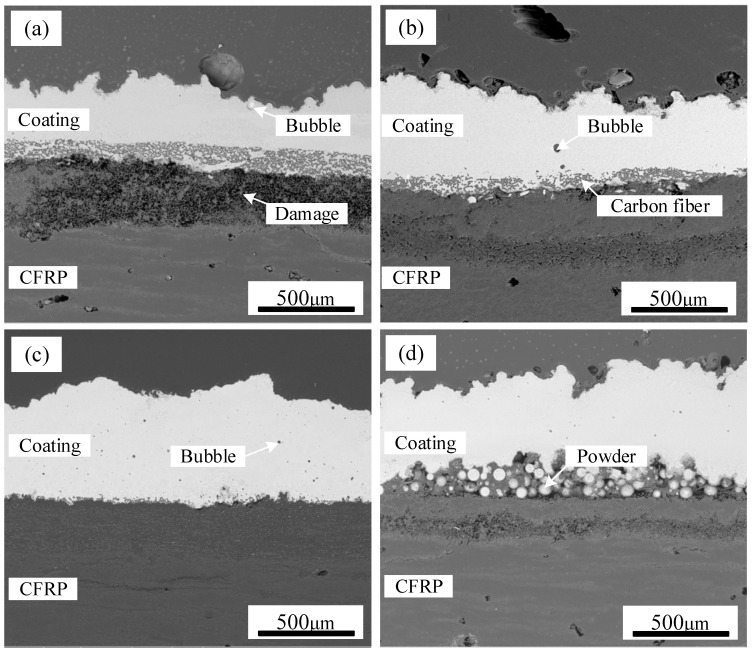
The cross-section of CFRP/TC4 under different scanning speeds: (**a**) v = 1 m/min, (**b**) v = 1.2 m/min, (**c**) v = 1.4 m/min, (**d**) v = 1.6 m/min.

SEM images of TC4 coating on CFRP deposited at different scanning speeds are displayed in Figure 8, highlighting the variations in coating integrity. When the scanning speed was set to 1 m/min, the substantial heat input caused severe thermal damage to the CFRP matrix near the interface. This excessive heat led to the detachment of numerous carbon fibers from the resin, significantly disrupting the original weaving pattern of CFRP. Consequently, an increased number of air voids remained within the TC4 coating, as seen in Figure 8a. When the scanning speed is elevated to 1.4 m/min, although enhanced coating uniformity is achieved, the penetration depth of TC4 into the CFRP substrate significantly diminishes. This reduction directly restricts the quantity of carbon fibers incorporated into the TC4 coating (Figure 8c), potentially compromising the interfacial bonding performance between the TC4 coating and CFRP. When the scanning speed reached 1.6 m/min, residual TC4 powder was observed at the CFRP/TC4 interface, as depicted in Figure 8d. This incomplete melting of the pre-positioned TC4 powder was attributed to the lower heat input at higher scanning speeds, which was insufficient to facilitate the formation of a continuous coating.

Overall, during the laser cladding process for depositing TC4 coating on CFRP, the upper TC4 powder layer is subjected to intense localized heating by the laser beam, leading to its instantaneous melting. Due to temperature gradients within the molten pool, the liquid metal undergoes convective flow, either from the center outward or vice versa, to facilitate heat and mass transfer. As the temperature at the bottom of the TC4 powder layer rises, it eventually reaches the melting point of the matrix resin, initiating its thermal decomposition. As the laser beam progresses, the previously molten area undergoes rapid cooling, leading to nucleation and crystallization, which ultimately form the solidified TC4 coating. Throughout this process, the surface tension of the molten TC4 metal induces spheroidization, which contributes to surface irregularities in the final coating. Therefore, optimizing processing parameters is essential to achieving a continuous and uniform TC4 coating on the CFRP substrate, ensuring enhanced coating quality and performance.

### 3.3. Microstructure

**Figure 9:**
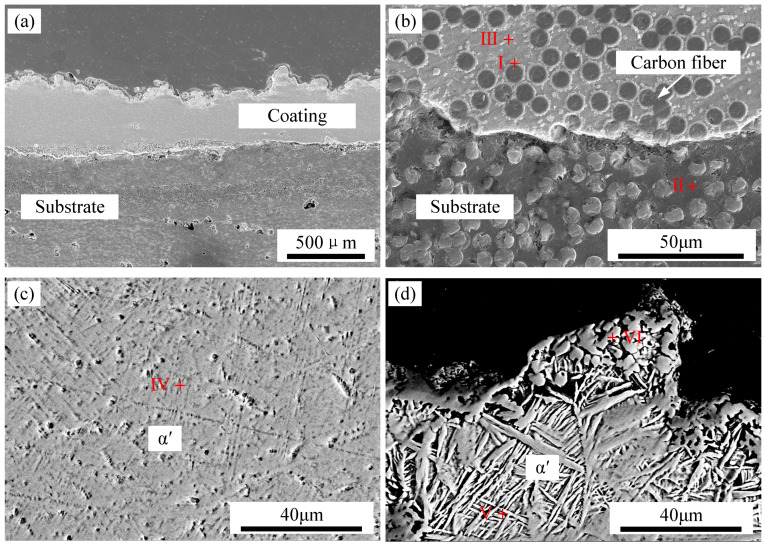
Section micromorphology of TC4 coating on CFRP: (**a**) overall appearance, (**b**) interface of coating, (**c**) middle of coating, (**d**) top of coating.

SEM was employed to examine the microstructure and morphology of TC4 coating on CFRP produced by laser cladding. Figure 9 presents SEM images of a TC4 coating fabricated under a laser power of 250 W and a scanning speed of 1.2 m/min, while Table 2 provides the energy-dispersive spectroscopy (EDS) results for regions I–V. The overall coating morphology is depicted in Figure 9a, whereas Figure 9b focuses on the interface region between the TC4 coating and the CFRP substrate. The results indicate that TC4 infiltrated the CFRP gaps, forming a well-bonded interface devoid of pores or cracks.

The formation of intermetallic phases at the interface is influenced by the electronegativity values of Ti (1.54), V (1.63), Al (1.61), S (2.58), and C (2.55), which correlate with the stability of the resulting compounds. Consequently, Ti-C and Ti-S phases are preferentially formed in the IMC layer. At position I within this layer, the Ti content was 51.11 at.%, while the C content was 40.2 at.%. According to the Ti-C binary phase diagram, Ti and C readily react to form TiC, suggesting that TiC is the dominant phase in the IMC layer. The microstructural morphology of the central region of the TC4 coating is displayed in Figure 9c. Here, a fine, short acicular structure is observed, with EDS analysis at position IV revealing a Ti content of 81.08 at.% and a C content of 4.51 at.%. This composition suggests the presence of a mixed microstructure consisting of acicular α′ martensite and TiC. The uppermost microstructure of the TC4 coating is shown in Figure 9d, where a combination of granular and basket-like phases is evident. EDS analysis at position VI identifies a Ti content of 53.5 at.% and a C content of 37.19 at.%, confirming the presence of TiC in the granular phase. Additionally, at position V, the Ti content was measured at 79.24 at.% and the C content at 6.47 at.%, indicating a composite microstructure of TiC and acicular α′ martensite. The TiC microstructure predominantly exhibits layered, granular, rod-like, and dendritic morphologies. The acicular α′ martensite in this region is characterized by a high aspect ratio. The formation of the basket-like structure is attributed to lower heating temperatures or rapid cooling rates, which increase the nucleation rate of α′ martensite in various orientations, resulting in smaller α′ clusters, shorter and broader α′ bars, and an intertwined basket-like arrangement dominated by acicular α′ martensite.

### 3.4. Structure of the Interfacial Phase

**Figure 10:**
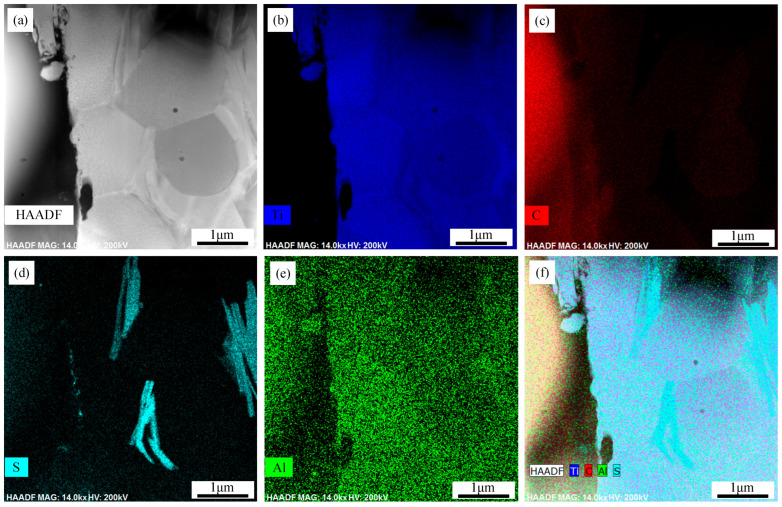
TEM analysis of CFRP/TC4 interfacial FIB sample: (**a**) HAADF image, (**b**) Ti distribution, (**c**) C distribution, (**d**) S distribution, (**e**) Al distribution, (**f**) composite image.

To elucidate the binding mechanism between the TC4 coating and the CFRP substrate, TEM was employed to analyze the interface between these two materials. The interface was sectioned at the junction of the TC4 coating and the carbon fibers within the CFRP. Figure 10a displays the backscattered morphology of the cut FIB specimen, where the black region on the left corresponds to the carbon fibers, while the gray–white region on the right represents the IMC layer. The results indicate the formation of a strong bond between the CFRP and the TC4 coating, characterized by a jagged interface. The IMC layer consists of large polygonal grains with an irregular morphology and an approximate grain size of 2 μm.

As depicted in Figure 10b–e, the FIB specimen contains Ti, Al, C, and S, whereas the original TC4 powder lacks both C and S. This observation confirms that a chemical reaction occurs at the interface between the TC4 powder and the CFRP matrix, facilitating atomic diffusion. Analysis of the elemental distribution reveals that Ti and Al are homogeneously dispersed throughout the IMC layer, with no noticeable segregation. Carbon is predominantly concentrated within the large polygonal grains, while sulfur is located at the grain boundaries, forming striped structures within these regions. Based on these findings, it can be concluded that metallurgical bonding between the TC4 coating and the CFRP matrix is achieved through the combined effects of laser energy and reaction heat, promoting interfacial diffusion and compound formation.

### 3.5. Mechanical Performance and Surface Morphology of Fractures

**Figure 11:**
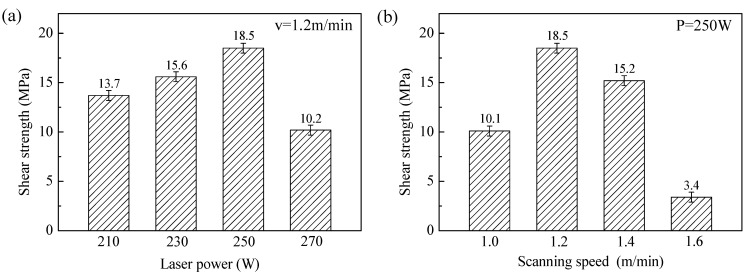
Influence of laser process parameters on interface bonding performance: (**a**) different laser power, (**b**) different scanning speed.

A compression shear test was conducted to evaluate the impact of laser processing on the interface bonding properties of the TC4 coating on CFRP, which was prepared using the laser cladding method. Figure 11 presents the shear strengths of samples processed under various laser power settings. Figure 11a illustrates the variation in interface strength with laser power at a scanning speed of 1.2 m/min. As the laser power increased, the bond strength of the CFRP/TC4 interface initially increased before decreasing. The maximum shear strength observed at the interface was 18.5 MPa, a significant improvement over coating prepared by spraying. This increase in strength is primarily attributed to the formation of chemical bonds at the interface, and metallurgical bonding is achieved.

Figure 11b shows the relationship between interface strength and scanning speed at a fixed laser power of 250 W. As the scanning speed increased from 1 m/min to 1.6 m/min, the binding strength at the CFRP/TC4 interface first increased, then decreased. It is noteworthy that no significant shear strength reduction occurs at the scanning speed of 1.4 m/min. Analysis of the SEM results (Figure 8c) reveals limited metallurgical bonding between TC4 and CFRP under this parameter. A plausible explanation lies in the laser-induced thermal decomposition of CFRP, which generates gas bubbles that solidify into porous cavities during cooling. The TC4 cladding layer subsequently infiltrates these cavities, establishing a mechanical interlocking mechanism that compensates for weak metallurgical bonding and enhances substrate–coating adhesion. This phenomenon suggests that strategically increasing laser power to improve metallurgical interaction at 1.4 m/min scanning speed could synergistically optimize the TC4/CFRP interfacial performance. At a scanning speed of 1.6 m/min, the shear strength dropped to only 3.4 MPa. Based on the micromorphology analysis of the cross-section of the cladding layer, it is evident that these results suggest that excessively high laser power or low scanning speed results in increased heat input. This causes the laser energy to transfer into the CFRP side, leading to interfacial damage, ablation, and the formation of cracks, pores, and other defects.

**Figure 12:**
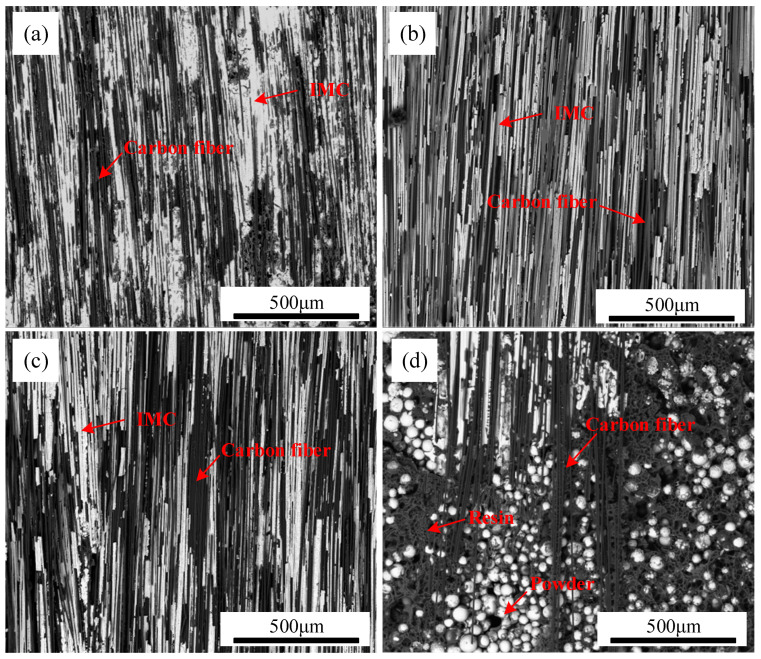
Microstructure of the coating side of the compression shear fracture at different scanning speeds: (**a**) v = 1 m/min, (**b**) v = 1.2 m/min, (**c**) v = 1.4 m/min, (**d**) v = 1.6 m/min.

Figure 12 presents the surface morphology of the CFRP side of the compression fracture for samples prepared at different scanning speeds, where the laser power was set at 250 W. When the scanning speed ranged from 1 m/min to 1.6 m/min, the fracture surface appeared rough, exhibiting exposed carbon fibers and strip-like grooves. The fractures occurred at the interface between the coating and the carbon fibers. At a scanning speed of 1.6 m/min, a significant area remained as non-melted TC4 powder (Figure 12d). This poor bonding between the TC4 powder and the CFRP matrix resulted in weak compression shear performance, confirming that the CFRP/TC4 interface was the weak point. The primary cause of this phenomenon is the large thermal property differences between CFRP and the coating, which generate substantial residual stress at the CFRP/TC4 interface. This residual stress increases as heat input rises, leading to a reduction in interfacial bonding strength. Therefore, in the cladding process, it is recommended to use lower laser power or higher scanning speeds to minimize heat input.

**Figure 13:**
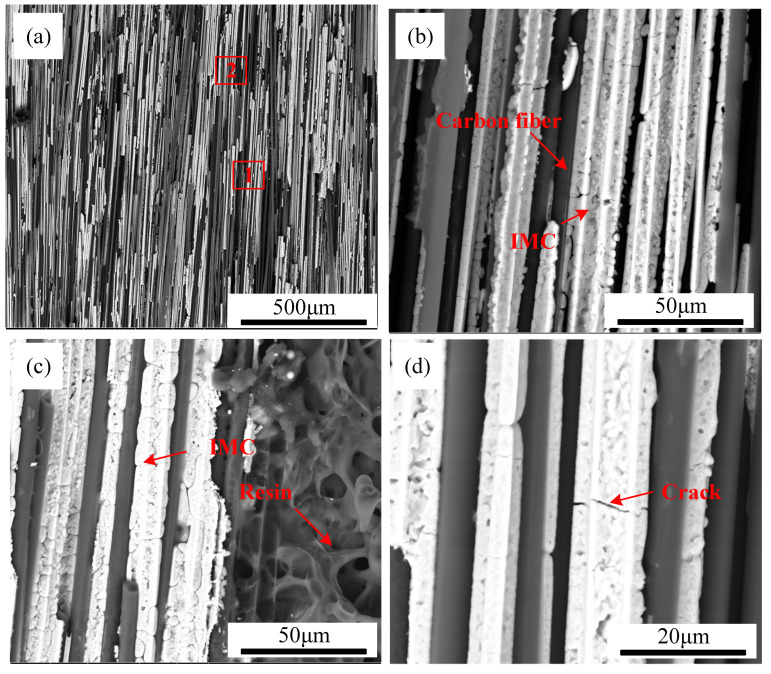
Fracture surface of the compression shear sample on the TC4 coating side (P = 250 W, V = 1.2 m/min): (**a**) surface morphology, (**b**) magnified view of area 1, (**c**) magnified view of area 2, and (**d**) crack morphology.

Figure 13 shows the surface morphology of the CFRP side of the compression fracture for TC4-coated CFRP samples prepared by laser cladding with P = 250 W and v = 1.2 m/min. The fracture surface displayed numerous striped grooves, uneven topography, and high roughness. A substantial amount of carbon fibers and some resin remained attached to the silver-white TC4 coating, suggesting that the fracture mechanism was cohesive failure. Figure 13b and Figure 13c provide high-magnification images of areas 1 and 2 in Figure 13a, respectively, where exposed carbon fibers and stripe-like grooves are evident. These grooves primarily form due to the interaction between TC4 and CFRP, resulting in the formation of IMC at the interface. Under compressive shear force, carbon fibers at the CFRP/TC4 interface detach from the IMC, creating the observed stripe-like grooves. Cracks within the IMC layer were also observed (Figure 13d). It is worth noting that unmelted powders may also cause local weaknesses, resulting in stress concentrations and facilitating the onset of cracks during shear testing. All of this indicates that fracture occurs predominantly at the interface between the IMC and the carbon fibers.

**Figure 14:**
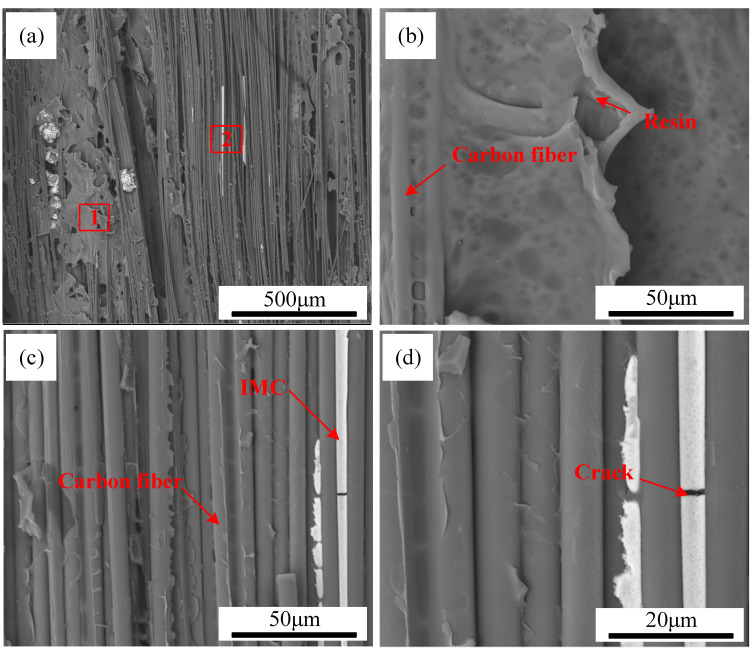
Fracture surface of the compression shear sample on the CFRP side (P = 250 W, V = 1.2 m/min): (**a**) surface morphology, (**b**) magnified view of area 1, (**c**) magnified view of area 2, and (**d**) crack morphology.

Figure 14 presents further surface morphology details of the CFRP side of the compression fracture for TC4-coated CFRP samples prepared by laser cladding. Figure 14a reveals a rough fracture surface. Figure 14b shows that PPS (polyphenylene sulfide) filled the spaces between carbon fibers, while Figure 14c highlights the presence of many exposed carbon fibers. Residual IMC was observed between the carbon fibers, and cracks were noted in the IMC layer (Figure 14d).

## 4. Conclusions

In this paper, laser cladding has proven to be an effective method for rapidly forming a continuous, uniform TC4 coating on the surface of CFRP, enabling the establishment of a chemical metallurgical bond between the TC4 coating and the CFRP matrix. Real-time monitoring of the cladding process using a high-speed camera system, along with SEM and TEM analyses of the morphology, combined with the interface shear strength and fracture morphology, allowed for the exploration of the relationship between “process, structure, and performance”. These findings provide both theoretical foundations and practical protocols for producing TC4 coatings with enhanced mechanical integrity and minimized CFRP substrate degradation, thereby accelerating the aerospace implementation of CFRP components with metallic protective surfaces. The following conclusions can be drawn:

(1) The real-time monitoring of the cladding process and the results from the joint interface formation reveal that when laser power is too low or the scanning speed is too high, the laser energy is insufficient to fully melt the TC4 powder, preventing the formation of a continuous coating. Conversely, when laser power is excessively high or the scanning speed is too low, thermal decomposition occurs, leading to the formation of cracks, pores, and other defects at the interface due to the poor thermal resistance of CFRP. Additionally, the gases produced by the thermal decomposition of PPS cause a strong impact on the coating, increasing surface roughness.
(2) In the CFRP/TC4 coating, the primary phases include granular TiC, acicular α′ Martensite, and a small amount of TiS_2_ at the interface. A continuous interface between the carbon fibers, resin, and coating was observed, confirming the chemometallurgical bonding between the TC4 coating and CFRP matrix under the combined influence of laser energy and interfacial reaction heat.
(3) The mechanical performance of the optimized TC4 coating, prepared on the CFRP surface with a laser power of 250 W and a scanning speed of 1.2 m/min, resulted in an interfacial shear strength of 18.5 MPa. During the compression shear test, the fracture of the interface between the coating and matrix occurred predominantly between the IMC and the carbon fibers, with the fracture mechanism identified as cohesive failure.

## Figures and Tables

**Table 1:** Chemical composition of TC4.

Fe	C	N	Al	V	O	Ti
0.18	0.02	0.05	6.51	4.13	0.185	bal.

**Table 2:** EDS results of marked areas in Figure 9 (at.%).

	Elements	C	Al	V	Ti
Area					
I		40.2	6.37	2.32	51.11
II		93.87	-	6.13	-
III		7.21	10.04	3.84	78.91
IV		4.51	10.78	3.63	81.08
V		6.47	10.7	3.59	79.24
VI		37.19	6.65	2.66	53.5

## Data Availability

Data are contained within the article.
